# Evaluation of P16 expression in canine appendicular osteosarcoma

**DOI:** 10.1186/s12917-017-1113-5

**Published:** 2017-06-20

**Authors:** B. G. Murphy, M. Y. Mok, D. York, R. Rebhun, K. D. Woolard, C. Hillman, P. Dickinson, K. Skorupski

**Affiliations:** 1 Department Pathology, University of California, Davis, School of Veterinary Medicine, Microbiology and Immunology, Davis, CA 95618 USA; 2 Department of Surgical and Radiological Sciences, Davis, CA 95618 USA

**Keywords:** Canine, Osteosarcoma, P16, Immunohistochemistry, Tissue microarray

## Abstract

**Background:**

Osteosarcoma (OSA) is a common malignant bone tumor of large breed dogs that occurs at predictable anatomic sites. At the time of initial diagnosis, most affected dogs have occult pulmonary metastases. Even with aggressive surgical treatment combined with chemotherapy, the majority of dogs diagnosed with OSA live less than 1 year from the time of diagnosis. The ability to identify canine OSA cases most responsive to treatment is needed. In humans, OSA is also an aggressive tumor that is histologically and molecularly similar to canine OSA. The expression of the tumor suppressor gene product P16 by human OSA tissue has been linked to a favorable response to chemotherapy.

**Results:**

We identified an antibody that binds canine P16 and developed a canine OSA tissue microarray in order to test the hypothesis that P16 expression by canine OSA tissue is predictive of clinical outcome following amputation and chemotherapy. Although statistical significance was not reached, a trend was identified between the lack of canine OSA P16 expression and a shorter disease free interval.

**Conclusions:**

The identification of a molecular marker for canine OSA is an important goal and the results reported here justify a larger study.

## Background

Canine osteosarcoma (OSA) is an aggressive and highly metastatic tumor of bone and although it can occur at any age and in any dog breed, it is most often diagnosed in adult to older large to giant breed dogs. OSA is the most common canine skeletal tumor, accounting for approximately 80–85% of all bone tumors in dogs [1–3] and tends to occur at the most active metaphyseal regions of the appendicular skeleton. Relative to other skeletal sarcomas, canine osteosarcoma pursues a more rapid clinical course with earlier lung metastasis than either chondrosarcoma or fibrosarcoma of bone [4]. Early metastatic spread to the lungs contributes to the poor overall prognosis for canine OSA. Human OSA is histologically and molecularly similar to canine OSA [5–7] and both can be difficult to manage clinically [8, 9].

The current treatment of choice for canine OSA is surgical excision (involving limb amputation or limb-salvage surgery) followed by adjuvant platinum-based chemotherapy. Stereotactic radiation therapy has been reported as an alternative means to achieve local control [10, 11]. The survival time of canine appendicular OSA with adequate local control and without adjuvant chemotherapy is 119–175 days with a 12 month survival rate of only 11–21% [12]. In addition to surgery, platinum-based chemotherapy alone or in combination with doxorubicin has been shown to improve survival time [13]. A large study involving 470 dogs identified no difference in outcome between dogs with appendicular osteosarcoma treated with carboplatin vs. alternating carboplatin and doxorubicin [14]. Radiation therapy, bisphosphonates, and analgesics such as non-steroidal anti-inflammatory drugs and opioids can be utilized for palliative therapies. Reflecting owner choices, the use of palliative therapies alone generally result in relatively short survival times [12].

In an attempt to predict biological behavior, canine OSA has been categorized into a number of different histologic subtypes. Some studies have indicated that canine OSA histologic subtyping is not predictive of biological outcome [15] while others have indicated that specific OSA subtypes, such as telangiectatic or fibroblastic, may have negative or positive prognostic implications, respectively [2, 16]. Histological classifications have been frequently shown to be less informative than molecular phenotypes when applied to therapeutic outcome across a wide range of cancer types. A molecular biomarker that is predictive of canine OSA disease progression may assist prognostic determinations and treatment decisions.

Expression of the tumor suppressor gene product P16 by tumor tissue has recently been shown to significantly correlate with chemotherapeutic response in human osteosarcoma [9] and loss of P16 expression by OSA cells has been associated with decreased survival time in human patients with OSA [17–20]. Inactivation of tumor suppressor gene products like P16 is an important event in oncogenesis that may contribute to the development of canine OSA. Therefore, P16 expression has the potential to be a predictive signature of canine OSA tumor biology.

The tumor suppressor gene product P16 belongs to a family of cyclin-dependent kinase inhibitors. When bound to their respective cyclin dependent kinase, these proteins increase the inhibitory effects of the retinoblastoma tumor suppressor gene at the G1-S checkpoint, a ‘point of no return’ for cell division [21, 22]. In a normally regulated cell, growth-inhibiting signals result in P16 expression, blocking DNA synthesis and cell cycle progression [23]. Deletion or mutational inactivation of P16 can defeat the protective effect of the G1-S checkpoint, potentially leading to unregulated cellular division in a genetically damaged cell. Four of 6 previously reported canine OSA cell lines have been shown to have undetectable P16 protein or mRNA expression [23] and numerous human cancers are associated with P16 gene mutations, including osteosarcoma [21, 23].

We hypothesized that the presence or absence of P16 expression by canine OSA tissue is predictive of clinical outcome. In order to test this hypothesis, the identification of a P16 antibody that reliably binds canine P16 protein in immunoblot and immunohistochemistry (IHC) assays was needed. Utilizing a set of control human and canine tissues with known P16 expression patterns [24], we identified an anti-P16 antibody with appropriate binding affinity in both immunoblot and IHC assays. In addition, we developed a set of canine tissue microarrays comprised of retrospective case material from 33 dogs with appendicular OSA with known treatment regimens and long-term outcomes.

## Methods

### Pathology and clinical features

In this retrospective study, 33 excisional biopsies (amputations) diagnosed as canine appendicular OSA were utilized from patients enrolled in a randomized prospective study comparing the adjuvant use of single-agent carboplatin or alternating carboplatin and doxorubicin [13]. The inclusion criteria for the current study were the availability of sufficient high quality, formalin fixed, paraffin embedded tumor tissue and known treatment and clinical outcome data. Representative bone and soft tissue biopsy samples had been previously sectioned and fixed in 10% buffered formalin for a minimum of 48 h. Mineralized tissue was thinly sectioned and decalcified in 15% formic acid for 2–4 days, as needed. Tissue samples were routinely processed, cut into 4 μm thick sections, placed on positively charged glass slides and stained with hematoxylin and eosin according to routine protocols. All specimens were confirmed to be consistent with the diagnosis of osteosarcoma by a single pathologist (MYM) based upon published criteria [2]. Canine OSA tumors were subclassified as osteoblastic, chondroblastic, fibroblastic or mixed subtypes based upon published criteria [2].

### Tissue microarray

For each paraffin embedded tumor, optimal sites of the formalin-fixed paraffin embedded blocks for core sampling were identified. Characteristics of optimal sites included the following features: high cellularity, minimal necrosis, minimal hemorrhage and minimal matrix or bone deposition. The construction of the tissue microarray (TMA) was based upon a 2009 review by Parsons and Grabsh [25]. An Advanced Tissue Arrayer (model ATA-100, Chemicon International) was used to cut and insert canine OSA tissue core samples, in triplicate, into pre-cast paraffin blocks (Paraplast Plus, Sigma Aldrich). For the 33 canine cases, four canine OSA TMAs were constructed using a 9 × 6 grid pattern. Three, 2 mm diameter core biopsies from each donor tissue block, along with 2 positive control core biopsies (canine glioblastoma tissue) were semi-randomly arranged in the paraffin block while the outer circumferential border was generally comprised of negative control tissue (normal canine cerebrum or renal cortical tissue). To generate unstained paraffin sections, each assembled TMA was cut into 4 μm thick sections with a microtome and placed on positively charged glass slides. TMA sections were stained with either hematoxylin and eosin stains according to standard protocols or were further processed for P16 immunohistochemistry.

### Immunohistochemistry for P16

Immunohistochemistry (IHC) assays were performed on whole sections of the positive control tissue (canine glioblastoma), negative control tissue (normal canine brain tissue and canine renal cortical tissue) and the constructed tissue microarrays. IHC assays were performed on 4 μm thick, formalin-fixed, paraffin-embedded tissue sections, mounted on charged slides, and air-dried overnight at 37^o^ C. In order to ensure reproducible and homogeneous results between the IHC assays, a consistent development time and protocol was utilized.

Sections were deparaffinized through xylene to reagent alcohol, and treated with 0.3% hydrogen peroxide in methanol for 30 min. Sections were then rehydrated to water through graded reagent alcohols, and stabilized in 0.1 M Phosphate Buffered Saline, pH 7.4 (PBS). Antigen retrieval required exposure of sections to Dako Target Retrieval Solution, pH 9 (Dako, S2368) at 95^o^ C for 30 min, followed by a 20 min cool down at room temperature. Sections were blocked for 20 min in 10% normal horse serum in PBS. The primary antibody, a rabbit anti-P16 (SC-373695, F-8, Santa Cruz Biotechnology) diluted 1:100 in PBS-Tween 20 (0.02%) was applied for 1 h. All reagent incubations were at room temperature, and PBS-Tween 20 rinses occurred twice between reagent applications for 3 min each change. Envision + System-HRP (Dako, K4003) was applied for 30 min to label bound rabbit anti-P16 antibodies. The label was visualized with NovaRed for peroxidase (Vector, SK-4800). Sections were counterstained in Mayer’s Hematoxylin, air-dried and coverslipped. Non-specific background was evaluated with a duplicate section receiving diluent in place of the primary antibody.

The sub-cellular location of the P16 antigen was determined microscopically as nuclear, cytoplasmic or membranous. To determine the level of P16 expression, a semiquantitative scoring system was used based upon the percent of neoplastic cells expressing P16 in a 100× field of magnification: negative (0%), 1+ (<25%), 2+ (26–79%), 3+ (80–100%) (at 100× magnification the entire TMA biopsy section filled the field of view). The intensity of P16 staining was not scored. The semiquantitative ordinal scoring system was developed in accordance with recommended histopathologic principles [26]. For each canine OSA lesion on the TMA, one, two or three tissue biopsies were examined and scored for P16 expression, as described above (Table 1). For each case, a consensus P16 staining pattern was determined (1 of 1, 2 of 2, 2 of 3, or 3 of 3 sections). Discordant cases (1 of 2 sections) were re-reviewed by two anatomic pathologists (MYM and BGM) until a consensus could be reached. All IHC sections were examined in a blinded fashion where the pathologists were not aware of case signalment, diagnosis or outcome.

**Table 1:** Clinical and pathologic data for canine appendicular osteosarcomas

case	De/NDe	tx	sex	breed	tumor location	subtype	# sct	p16 exp
1	De	C/D	MC	Mix	Proximal tibia	C	2	1+
2	De	C/D	FS	GSD	Proximal radius	O	3	3+
3	NDe	C	MC	Lab	Proximal humerus	O	3	3+
4	NDe	C	FS	Grt Dane	Distal radius	C	3	2+
5	NDe	C	FS	Rott	Proximal tibia	O	3	neg
6	NDe	C	MC	Gold	Ulnar diaphysis	O	3	1+
7	De	C/D	MC	Mix	Distal radius	O	3	3+
8	De	C/D	FS	St. Bern	Distal tibia	O	3	3+
9	Nde	C	FS	Gold	Distal femur	O	3	2+
10	De	C	FS	Rott	Distal femur	O	3	3+
11	De	C	MC	Rott	Distal tibia	O	3	neg
12	NDe	C/D	FS	G Pyr	Distal tibia	O	3	2+
13	De	C/D	MC	Bernese MD	Distal radius	O	2	neg
14	NDe	C	FS	Rott	Distal radius	O	3	neg
15	De	C	FS	Mix	Distal femur	O	3	3+
16	NDe	C	FS	OESD	Distal radius	O	1	3+
17	NDe	C/D	FS	Curly C Ret	Proximal humerus	O	3	3+
18	De	C/D	FS	Rott	Proximal tibia	O	1	1+
19	De	C/D	FS	Mix	Distal radius	O	3	3+
20	NDe	C	FS	Lab	Proximal humerus	O	3	3+
21	NDe	C/D	MC	Grey	Prox humerus	O	1	2+
22	NDe	C/D	MC	Mix	Distal radius	F	3	2+
23	Nde	C/D	FS	GSD	Distal tibia	O	3	3+
24	Nde	C/D	FS	Malam	Distal radius	O	3	3+
25	De	C/D	FS	Rott	Distal femur	O	3	neg
26	De	C	MC	Mix	Proximal humerus	O	3	3+
27	NDe	C/D	FS	Mix	Distal tibia	O	3	neg
28	De	C/D	MC	Bernese MD	Distal femur	C	3	1+
29	De	C	FS	Leonberger	Distal radius	O	3	1+
30	De	C/D	MC	Ana Shep	Distal radius	O	2	2+
31	De	C	FS	Lab	Distal femur	O	3	2+
32	De	C	MC	GS Pointer	Proximal humerus	C	3	2+
33	De	C	M	Rhod Rback	Distal radius	M	3	1+

*Abbreviations*: De- decalcified section, NDe- non-decalcified section, tx- treatment, C- carboplatin, D- doxorubicin, MC- male castrated, FS- female spayed, GSD- German Shepherd dog, Lab- Labrador Retriever, Grt Dane- Great Dane, Rott- Rottweiler, Gold- Golden Retriever, St. Bern- Saint Bernard, G Pyr- Great Pyrenees, Bernese MD- Bernese Mountain dog, OESD- Old English Sheep dog, Curly C Ret- Curly Coat Retriever, Grey- Greyhound, Malam- Malamute, Ana Shep- Anatolian Shepherd, GS Pointer- German Shorthair Pointer, Rod Rback- Rhodesian Ridgeback, C- chondroblastic OSA, O- osteoblastic OSA, F- fibroblastic OSA, M- Mixed type OSA, # sct- number of P16 IHC sections examined

### Immunoblot

Control human and canine tissues were obtained from cell culture, surgical biopsy or necropsy as previously described [24]. Tissues know to express P16 (positive controls) included the SAOS2 human osteosarcoma cell line, a canine high grade oligodendroglioma (O8) and a canine grade IV astrocytoma/glioblastoma (GBM, G2); canine tissues previously demonstrated to not express P16 (negative controls) included a different canine GBM (G4), a high grade oligodendroglioma (O5) and normal canine cerebrum (NB) [24].

Briefly, cells and tumor samples were lysed in radioimmunoprecipitation assay buffer (RIPA) lysis buffer (Boston BioProducts, Worcester, MA, USA) with 1X Halt protease and phosphatase inhibitors (Thermo Fisher Scientific, Rockford, IL, USA) and kept on ice or stored at −80 C if not used immediately. Cell lysates were electrophoresed in 4–20% SDS Precast Polyacrylamide Gels (Expedeon) at 150 V for 45 min. The lysates were transferred overnight onto a PVDF membrane (BioRad) at 50 V in a 5 C cold room (PAGEgel Dual Run & Blot Vertical Mini-Gel System). The PVDF membrane was washed three times in tris-buffered saline with 0.1% Tween-20 (TBST), blocked for 1 h with 5% nonfat milk/TBST (blocking buffer), and subsequently washed 3 more times with TBST. The PVDF membrane was then incubated overnight at 5C with mouse monoclonal anti-P16 (1:500, SC-373695, F-8, Santa Cruz Biotechnology) diluted in blocking buffer. Membranes were then washed 3× and incubated with HRP conjugated-goat anti-mouse IgG antibody (Santa Cruz Biotechnology, IgG-HRP sc-2005) for 1 h at room temperature. Finally, membranes were washed 3×, incubated for 1 min with Pierce ECL reagent (Thermo Fisher Scientific, Catalog number 32106) and imaged using a FluorChem E digital imaging system (ProteinSimple).

### Analysis and statistics

Disease free interval (DFI) and survival were defined as described in Skorupski et al. [13]. The Kaplan-Meier method was used to estimate DFI and survival and the log-rank test was used to compare DFI and survival times between P16 expression groups. Statistical analyses were performed using commercial software (GraphPad Prism version 6.0f) and a *p*-value of <0.05 was considered significant. To determine if the decalcification process interfered with P16 detection, the putative correlation between P16 expression and decalcification was determined using Fisher’s exact test.

## Results

### Pathology and clinical features

The anatomic locations of the canine OSA tumors, in general, followed a predictable pattern reflecting the most active growth plates of the appendicular skeleton. There were two exceptions to this rule, in case 2, the OSA lesion was identified in the proximal radius while in case 6, the tumor was identified in the ulnar diaphysis (Table 1). Consistent with the tendency of OSA to occur in larger dogs, most of the dogs were large to giant breeds. Approximately equal numbers of patients received each chemotherapeutic protocol. The majority of the tumors were of the osteoblastic subtype (*n* = 27), with fewer numbers of chondroblastic (*n* = 4) and one case each of fibroblastic and mixed subtypes. Sections of the tissue microarrays stained with hematoxylin and eosin demonstrated histologically recognizable canine positive control (GBM), canine negative control (normal renal cortical or brain tissue) and canine osteosarcoma tissues (Figs. 2 and 3).

### Immunoblot

**Fig. 1:**
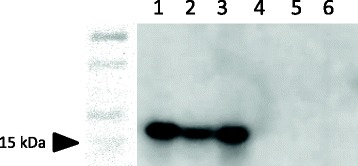
P16 antibody (F-8) binds human and canine P16 protein in a immunoblot assay**.** An appropriate size band (~15–16 kDa) is present in protein lysates derived from cells or tissues known to express P16 (lane 1- human osteosarcoma SAOS2; lane 2-high grade canine oligodendroglioma 08; lane 3- canine GBM G2). No bands are present in protein lysates derived from tissues known to not express P16 (lane 4 canine GBM G4; lane 5- canine oligodendroglioma; lane 6- normal canine brain NB)

Immunoblot assay with the primary anti-P16 antibody (F-8) revealed the presence of a 15-16 kDa band representing the P16 protein in the SAOS-2 human cell line (lane 1), a canine high grade oligodendroglioma (08, lane 2) and a canine GBM (G2, lane 3) (Fig. 1). No evidence of P16 protein expression was observed in negative control lanes 4–6 representing a different canine GBM (G4), a canine high grade oligodendroglioma (O5), and normal canine cerebrum (NB). These results were consistent with previous results using a different P16 antibody [24].

### Immunohistochemistry

**Fig. 2:**
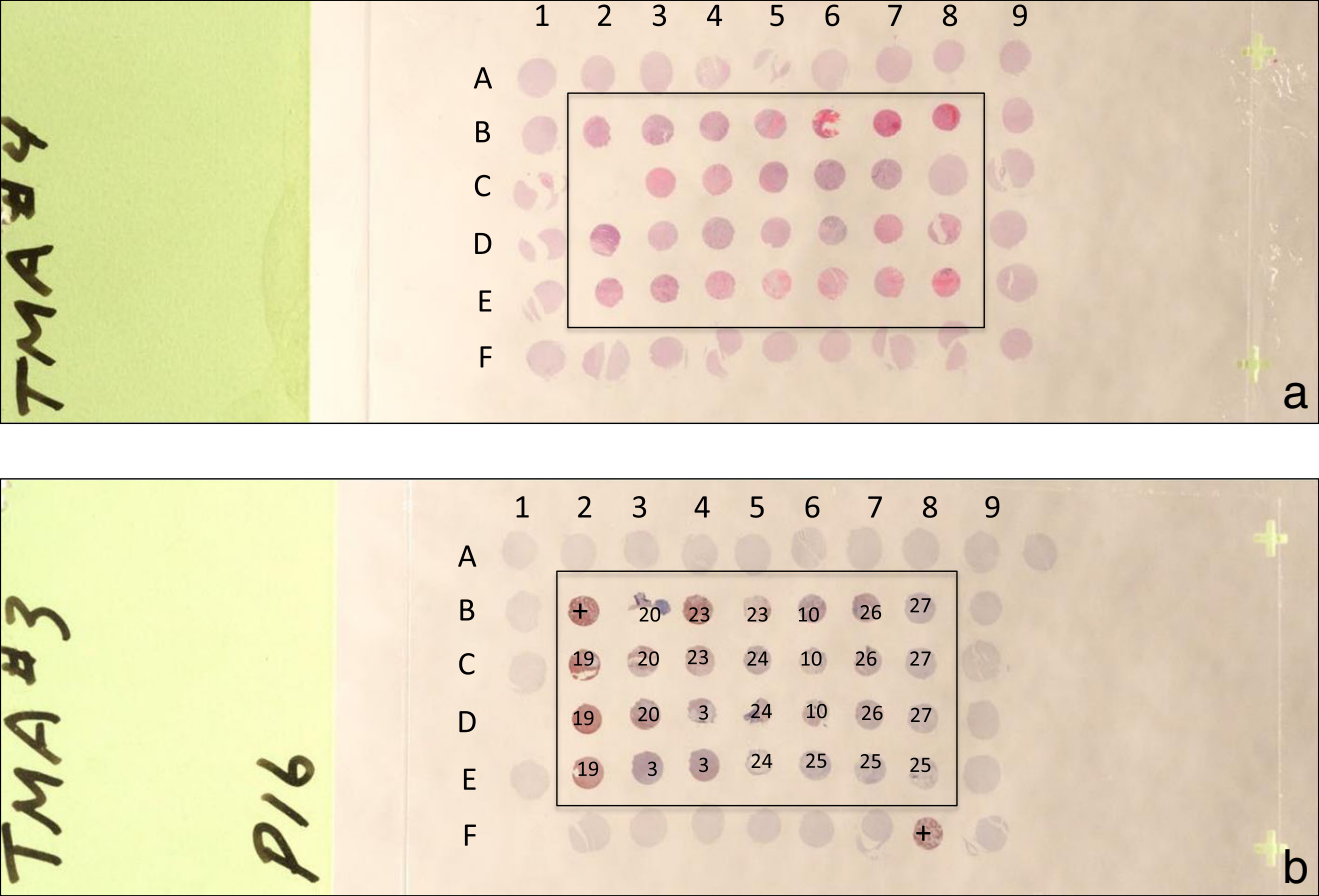
The organization and appearance of slides derived from canine OSA TMA stained with H&E stains or anti-P16 antibody (IHC). The TMA are arranged in 9 columns (1–9) by 6 rows (A-F) comprising a 9 × 6 grid. The majority of 2 mm diameter biopsy cores are present in hematoxylin & eosin-stained slides (TMA4) (**a**) and anti-P16 IHC (TMA3) (**b**). The outer row (columns 1 and 9, rows A and F) are generally comprised of negative control tissue (canine renal cortical or brain tissue). Triplicate canine OSA biopsy (test) samples are located within the boxed regions and are identified by case numbers (**b**). The location of positive control samples (GBM) are indicated (+) (**b**)

**Fig. 3:**
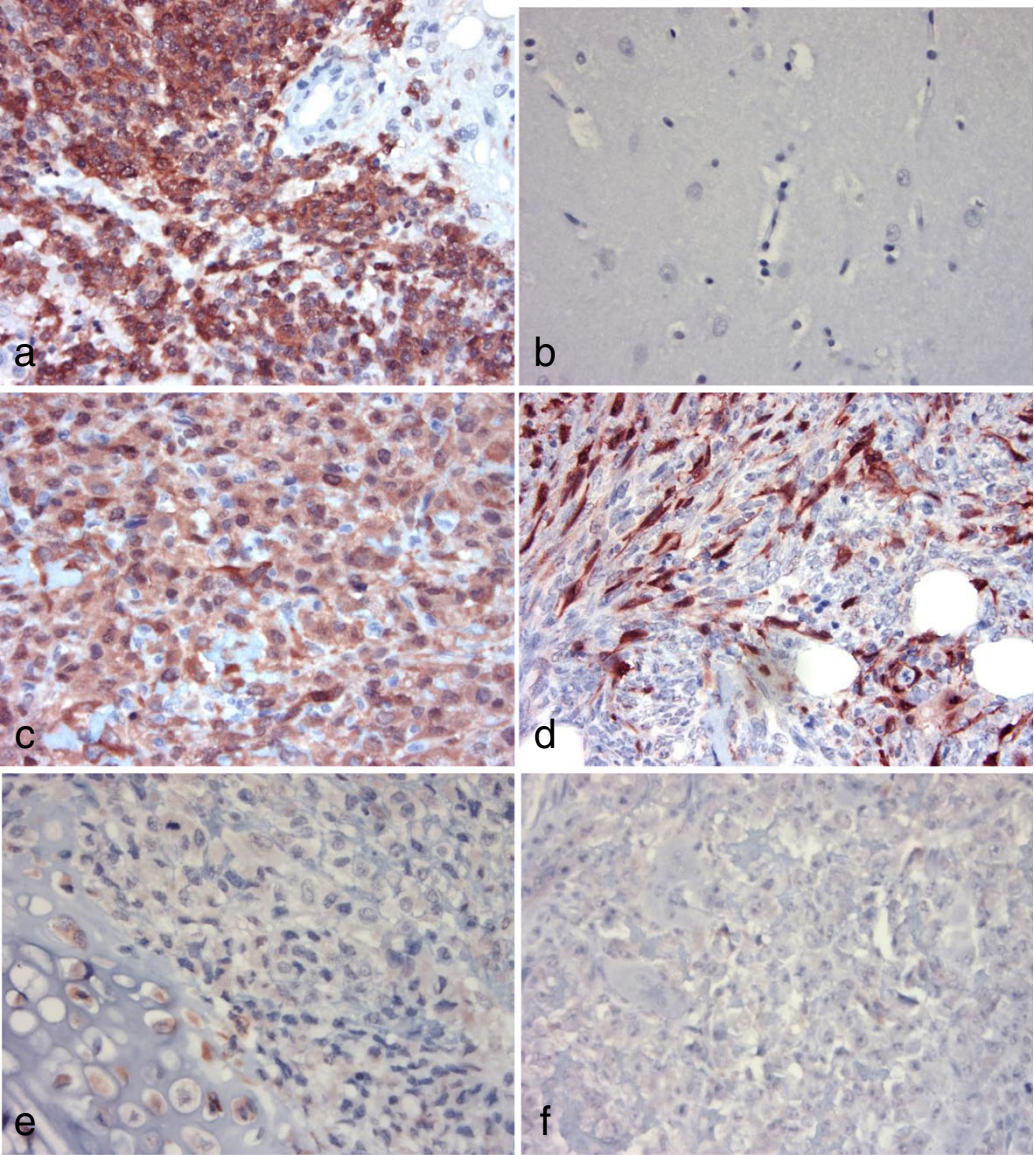
Immunohistochemistry assays reveal the proportion of neoplastic cells expressing P16. Canine glioblastoma cells demonstrate abundant *red-brown* staining in both the cytoplasm and nucleus (GBM, + control P16 IHC) (**a**). Normal canine brain tissue demonstrates an absence of *red-brown* stained cells (NB, negative control P16 IHC) (**b**). In a canine OSA, a majority of the neoplastic cells demonstrate red-brown cytoplasmic staining (3+ staining, case 19) (**c**). In a canine OSA, approximately 50% of the neoplastic cells demonstrate red-brown cytoplasmic staining (2+ staining, case 9) (**d**). In canine OSA, less than 25% of the neoplastic cells demonstrate red-brown cytoplasmic staining (1+ staining, case 4) (**e**). In canine OSA, none of the neoplastic cells demonstrate red-brown cytoplasmic staining (0+ staining, case 13). P16 immunohistochemistry, original magnification 400×

Using the same P16 antibody (F-8) as used in the immunnoblot assay, P16 immunoreactivity was noted within the cytoplasm and nuclei of the neoplastic cells comprising the canine GBM (G2, positive expression control) but was absent in normal canine cerebrum and canine renal cortical tissue (negative expression controls) (Figs. 2 and 3). Hence, an antibody capable of identifying the expression of P16 in IHC assays was identified.

OSA biopsy tissues in the TMA were assigned an ordinal value based upon the percent of neoplastic cells expressing P16 in a 100× magnification field: negative (0% cells), 1+ (<25% cells), 2+ (26–79% cells), 3+ (80–100% cells). In the majority of cases, three biopsy cores were examined and scored (*n* = 26 cases). In the remaining 6 cases, less than 3 biopsy cores were available for scoring as a result of loss during sectioning of the TMA blocks. In 3 cases, two biopsy cores were examined, and in 3 cases, a single core was examined and scored (Table 1). Although decalcification had no significant effect on the immunohistochemical scoring for P16 expression (*p* = 1.0) (Table 1), the effect of treatment or lack of treatment with decalcifying agents was not assessed within the same sample.

### Survival

**Fig. 4:**
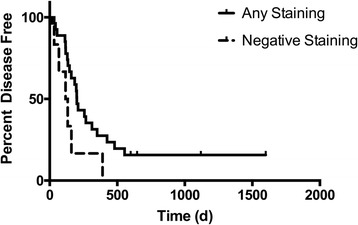
For dogs with OSA, the disease free interval (DFI) is shorter for tumors expressing P16 relative to tumors without P16 expression. The percent survival of dogs with OSA that exhibit *any* P16 staining (+1, +2 or +3, dashed line) and dogs with OSA that lack P16 staining (*solid line*) are plotted in this survival plot

Dogs with negative P16 immunoreactivity had a median disease free interval (DFI) of 125 days compared to 201 days for dogs with *any* evidence of P16 immunoreactivity (1+, 2+ or 3+ P16 immunoreactivity, Fig. 4). This difference approached statistical significance with a *p* value 0.055. Dogs with negative P16 immunoreactivity had a median survival time of 179 days compared to 353 days for dogs with any P16 immunoreactivity. This difference was not significant (*p* = 0.2). A comparison of samples with strong p16 immunoreactivity (+2 or +3) versus negative immunoreactivity was performed and found to be not significant (*p* = 0.09 for DFI and 0.3 for survival, respectively; data not shown).

Fourteen dogs treated with a combined chemotherapy regimen (carboplatin and doxorubicin) demonstrated at least some P16 expression while 3 dogs treated with a combined therapy lacked P16 expression. 13 dogs treated with carboplatin alone had P16 expression while 3 dogs treated with carboplatin alone lacked P16 expression (Table 1). As a result, P16 expression did not correlate with chemotherapy protocol (*p* = 1.00).

## Discussion

In this study, we identified an anti-P16 antibody that specifically labeled canine and human cells/tissues previously shown to express P16 protein in immunoblots and immunohistochemistry assays (positive controls); and failed to label cells and tissues known to not express P16 protein (negative controls). The Santa Cruz F-8 antibody is directed towards an epitope mapping between amino acids 4–31 at the N-terminus of human P16. Within this region, there are 4 amino acid mismatches between the human and canine P16 protein sequence (data not shown). This difference between the human and canine P16 sequences is apparently insufficient to abrogate antibody binding.

Using this antibody in IHC assays with a set of 4 canine OSA tissue microarrays demonstrated P16 expression varying from no expression (0% of the neoplastic cells express P16), up to 3+ expression (80–100% of the neoplastic cells express P16). Although statistical significance was not reached, a trend was identified between the lack of P16 expression (negative staining) and a shorter DFI. This finding in canine OSA is intriguing as inactivation of P16 expression by mutation, deletion, or promoter hypermethylation has been associated with continuous cell proliferation in human OSA [9] and loss of P16 expression has been correlated with decreased survival time in human OSA [17].

In all of the examined canine OSA biopsy sections, P16 protein expression, when present, was determined to be cytoplasmic. In the positive control tissue (canine GBM), P16 expression was identified in both the nucleus and cytoplasm of the neoplastic cells. Although the specificity and validity of cytoplasmic localization of P16 observed in some tumors has been questioned, P16 has been shown to be expressed in the cytoplasm of a wide variety of cell types [27–29]. Studies have reported the localization of P16 protein in both the nucleus and the cytoplasm in multiple neoplasms.

Interestingly, four of the six dogs with no P16 expression were Rottweilers, while only two of the 27 dogs with some P16 expression were Rottweilers (Table 1). This finding is intriguing as Rottweilers, along with Greyhounds and Great Danes, have been shown to have an increased risk of developing OSA [30]. McNeill and co-workers found that relative to other dog breeds, Rottweilers are more likely to have an aggressive form of OSA with a higher likelihood of brain metastasis [31]. However, the McNeil study did not confirm that these differences were associated with a worse outcome. Studies have demonstrated an association between specific dog breeds, like Rottweillers, and the distribution of genomic copy number imbalances in canine appendicular osteosarcoma [32]. Such results indicate that individual genetic backgrounds, as defined by dog breed, influence tumor karyotypes in cancers like OSA with extensive genomic instability. Reconciling the findings reported here with these previous studies will require a larger population of animals.

This pilot study had several limitations. Due to sporadic sectional loss in the TMA, three biopsy cores were not available for examination for every case. The concordance for IHC staining between tissue arrays with triplicate cores per tumor and full sections has been shown to be 96–98% [33]. In the study described here, the majority of cases (*n* = 26) had three examined biopsy cores. However, as a result of tissue loss in some of the TMA sections, only two biopsy cores were examined for 3 cases, and a single core was examined and scored for 3 cases. The percent mismatch (nonconcordance) between the immunohistochemical scoring of biopsy cores and the full section has been determined to be 3.7, 4.4 and 9.4% for 3, 2 and 1 core, respectively [33]. Although the examination and scoring of less than 3 sections is considered suboptimal, a nonconcordance rate of <10% was considered to be acceptable for this pilot study.

Overall case numbers were low (*n* = 33), and the number of cases completely lacking P16 expression (*n* = 6) was particularly low. This limitation might have resulted in a type II error (“false negative”) in results comparing P16 staining to overall outcome (DFI). This possibility is suggested by the close *p*-value for DFI and large difference in median survival despite a non-significant *p*-value. In addition, dogs included in the study received 2 different adjuvant chemotherapy protocols and the study from which the cases were collected showed a significant difference between DFI in these protocols. Although we found no association between P16 staining and chemotherapy protocol, larger studies with uniform treatment would be more ideal.

## Conclusions

This study, demonstrating a trend between the lack of P16 expression in canine appendicular OSA lesions and a shorter DFI, provides preliminary data justifying a larger study. The identification of a molecular marker reliably indicating prognosis for canine appendicular OSA is needed.

## Abbreviations

DFI: Disease free interval
GBM: Glioblastoma
IHC: Immunohistochemistry
NB: Normal canine cerebrum
OSA: Osteosarcoma
PBS: Phosphate buffered saline
TMA: Tissue microarray
